# Effect of high-starch or high-fibre diets on the energy metabolism and physical performance of horses during an 8-week training period

**DOI:** 10.3389/fphys.2023.1213032

**Published:** 2023-09-08

**Authors:** Agathe Martin, Romuald Lepers, Maximilien Vasseur, Samy Julliand

**Affiliations:** ^1^ Lab To Field, Dijon, France; ^2^ INSERM UMR 1093-CAPS, Université de Bourgogne, UFR des Sciences du Sport, Dijon, France

**Keywords:** French Trotters, incremental test, gas exchange, VܩO_2_, VܩCO_2_, alfalfa

## Abstract

Large amounts of high-starch concentrates are traditionally fed to horses in training. However, this has been associated with digestive or muscle diseases and behavioural modifications. In parallel, it has been demonstrated that horses fed high-fibre, low-starch diets achieve the same performance over an exercise test as the ones fed high-starch diets. However, whether the same performance level can be maintained over a longer training cycle is still being determined. This study aimed to compare the evolution in physical performance and cardiorespiratory responses of two groups of French Trotters fed either a control high-starch (15.0 g dry matter hay/kg body mass/day + 6.6 g dry matter oats/kg body mass/day) or a high-fibre diet (75% of oats replaced by dehydrated alfalfa) over an 8-week training period. The horses that entered the trial were untrained for ≥4 months and previously fed hay only. Track training with speed monitoring included interval training sessions and 2400 m performance tests from week 1 to week 8 (W8). Before (week 0, W0) and after (week 9, W9) the training period, horses performed an incremental continuous exercise test during which cardiorespiratory parameters were measured. Both groups progressed to the same extent regarding physical performance measured during interval training sessions (acceleration: 0.16 m.s^−2^ at W0 and 0.40 m.s^−2^ at W8; *p* < 0.0001), the 2400 m performance test (average speed: 8.88 m.s^−1^ at W0 and 10.55 m.s^−1^ at W8; *p* < 0.0001), and the incremental continuous exercise test (speed during the fastest stage: 9.57 m.s^−1^ at W0 and 10.53 m.s^−1^ at W9; *p* = 0.030). Although oxygen consumption increased with training (*p* = 0.071), it was not influenced by the diet. On the contrary, carbon dioxide production increased in the high-starch group only (high-starch group: 84.0 vs. high-fibre group: 77.7 mL.kg^−1^.min^−1^ at W9; *p* = 0.031). The results illustrate that horses in both groups progressed similarly but did not use the same metabolic pathways during exercise. This hypothesis is supported by carbohydrate oxidation, which tended to increase in the high-starch group at W9 but decreased in the high-fibre group (*p* = 0.061). In conclusion, the substitution of high-starch by high-fibre diets enabled similar performance over an 8-week training period and altered energy metabolism in a way that could be beneficial during high-intensity exercise.

## 1 Introduction

The physical activity of horses leads to increased energy requirements. Compared with basal situation, a doubling of energy requirements is estimated in horses working at “very heavy exercise” (National Research Council, 2007). To satisfy this increase in energy requirements, racehorses typically receive an average of 5–7 kg of cereals or concentrate feeds per day, rich in starch and simple sugars (Richards et al., 2006; Sommaire, 2007; Slove, 2011; Kaya-Karasu et al., 2018). However, high intakes of starch and simple sugars in horses lead to health risks and have been associated with gastric (Luthersson et al., 2009), intestinal (Collinet et al., 2021), and muscle diseases (McKenzie, 2017), as well as behavioural modifications (Bulmer et al., 2015; Destrez et al., 2019).

Horses are physiologically adapted to ingest and digest high-fibre diets. The microbiota hosted in their large intestine is effective in ensuring the digestion of cellulose, hemicelluloses, and pectins which constitute plant cell walls (Julliand and Grimm, 2017). This leads to a massive production of volatile fatty acids (VFAs), mostly composed of acetate, representing more than two-thirds of the fermentation end-products (De Fombelle et al., 2003). In horses, VFAs naturally represent the first source of energy (Argenzio, 1975). Glucose originating from starch hydrolysis in the small intestine is absorbed through intestinal mucosa which significantly increases glycaemia (Rodiek and Stull, 2007), and digestion of lipids leading to postprandial increased blood fatty acids is highly efficient in horses (Kronfeld et al., 2004). Equine muscle cells can utilise acetate, glucose, and lipids for their oxidative energy metabolism.

During short, high-intensity exercise, lipids contribute little to the metabolism of horse muscle cells, unlike glucose (Hodgson, 1985; Lawrence, 1990). Acetate is a direct precursor of acetyl-CoA, which reacts in the first step of the Krebs cycle under aerobic conditions. Utilising the arteriovenous difference, it has been measured that 39% of acetate is extracted from arterial blood entering the hindlimb of Thoroughbreds, regardless of the blood concentration (Pethick et al., 1993). In this same study, it was measured that acetate contribution to oxidative metabolism in the hindlimb could rich 32% when the horses were fed a 100% forage diet and 21% when the diet consisted of half forage and half oats, which disturbs fibrolysis in the large intestine (Pethick et al., 1993). The use of acetate as an important energy source in horses during exercise is subsequently confirmed. A drop in blood acetate concentrations is observed in Standardbred geldings between the beginning and the end of their warm-up and between the beginning and the end of a continuous incremental exercise test on a treadmill (Jansson and Lindberg, 2012). The decrease is more pronounced in horses fed a high-fibre diet, which initially have the highest blood acetate concentrations. This suggests that skeletal muscles utilise available energy substrates and that acetate is rapidly mobilised when abundant. After an exercise [2,600 m at 90% of maximal oxygen consumption ( $\dot{V}O_{2\max}$ ) on a 2.5% incline], muscle glycogen depletion and lactataemia are lower when Standardbred geldings are fed a high-fibre diet than a high-starch diet, suggesting that the use of acetate could reduce the glycolytic pathway (Karlsson et al., 2002). These elements assume that a high-fibre, low-starch diet can occasionally allow horses to achieve the same level of performance as a “traditional” high-starch diet fed in racing stables. However, it is not known if a similar level of performance could be maintained during a more extended training period with a high-fibre, low-starch diet. Therefore, the first objective of this study was to compare the changes in performance between two groups of horses, fed either a high-fibre or a high-starch diet, over an 8-week training period. To produce acetyl-CoA and fuel the Krebs cycle, hydrolysis of glucose to pyruvate and beta-oxidation of lipids use dehydrogenases, contrary to acetate. This mechanism produces protons, the elimination of which increases the amount of exhaled CO_2_ (Péronnet and Aguilaniu, 2006). We hypothesised that the energy metabolism of horses fed a high-fibre, low-starch diet would lead to changes in CO_2_ production ( $\dot{V}{CO}_{2}$ ) during exercise. The second objective of this study was, thus, to compare the cardiorespiratory responses to exercise of horses fed a high-fibre low-starch or a high-starch diet.

## 2 Materials and methods

### 2.1 Ethics

All methods and procedures used in this study were evaluated and approved by the institutional ethics committee (Comité d’Ethique de l’Expérimentation Animale Grand Campus Dijon). The project was authorised by the French Ministry of Higher Education, Research, and Innovation (registration number APAFIS#24932-2020040120076490 v2).

### 2.2 Experimental design

In total, 10 healthy gelding French Trotters [5–11 years old, 537 ± 33 kg body mass (BM)] retired from race training and untrained for at least 4 months before the beginning of the trial were included in the study. Horses were stabled in individual boxes and were allowed 2.5 h free exercise daily in paddocks. After 5 weeks (W4–W0) of habituation to the stable management, the horses completed an initial incremental continuous exercise test (ICET) to determine their maximal aerobic speed (MAS) before being allocated into two homogenous groups in terms of age, weight, body condition score, MAS, $\dot{V}O_{2}$ , and $\dot{V}{CO}_{2}$ measured during the basal ICET. Apart from the diet, management was then similar between the groups, and horses completed an eight-week longitudinal trial (from W1 to W8), ending with a second ICET the week after (W9).

### 2.3 Animal management and diet

#### 2.3.1 Training

**FIGURE 1:**
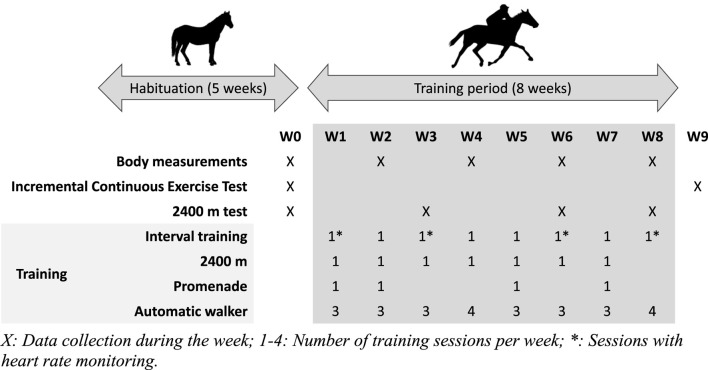
Repartition of training sessions, exercise, tests, and measurements throughout the study.

Horses were trained 6 days a week, either during ridden sessions or with an automatic walker, according to a schedule defined before the trial (Supplementary Table S1). The training load was adapted each week to increase regularly in volume and intensity. Depending on the training week, horses were ridden two or three times and exercised three or four times in a walker (60 min at 1.7 m.s^−1^). All horses performed the same number of training sessions during the trial. The ridden work consisted of three different exercises, alternating during the week after an initial warm-up: “interval training” (IT—4 to 8 maximal accelerations from 5.0 m.s^−1^ to peak velocity at trotting on approximately 150 m in a straight line on a track), “2,400 m” (one or two sequences of 2,400 m on a track at 70% or 80% of the basal MAS), and “promenade” (5200 m at 60% of the basal MAS on a path). Every third week, the “promenade” training was replaced by a 2,400 m performance test on the track, where horses had to cover 2,400 m at their maximal speed (Figure 1). During the 8 weeks of training, all horses were trained in an equal number of sessions for each type of exercise, and every kind of exercise was led by the same jockey for all the horses.

#### 2.3.2 Diets

During the habituation period, horses were fed hay only to meet 100% of their energy requirements at maintenance (National Research Council, 2007). During the training period, five horses were fed the diet “Control” (CON), and five the diet “Alfalfa” (ALF). These two diets were individually calculated at the beginning of the training period and maintained all along the trial. Expressed in dry matter (DM), the diet CON was composed of 68% hay and 32% oats, and the diet ALF 60% hay, 7% oats, and 33% dehydrated alfalfa (mix of 70% long strands and 30% pellets) (Table 1). Hay, oats, and alfalfa were analysed by traditional wet chemistry procedures (Dairy One Laboratory, Ithaca, NY, United States) to determine biochemical composition. Both CON and ALF diets were formulated to meet 100% of the energy requirements for horses subjected to heavy work and to provide 100% of the estimated protein requirements for the diet CON and 130% for the diet ALF based on National Research Council (2007). Total DM intake was 2.2 g DM/kg BM/day for the diet CON and 2.5 g DM/kg BM/day for the diet ALF. Hay and concentrates were offered simultaneously, in two equal meals daily at 08:00 and 16:30. Each horse had free access to a NaCl block and to an automatic waterer.

**TABLE 1:** Chemical and nutritional composition of the diets.

	Control diet	Alfalfa diet
Diet composition (g DM/kg BM/day)		
Meadow hay	15.0	15.0
Whole oats	6.6	1.7
Dehydrated alfalfa	—	7.8
Nutritional composition of the diet		
Digestible energy (kJ/kg BM/day)	222.93	222.27
Crude protein (CP, g/kg BM/day)	1.75	2.32
Starch (g/kg BM/day)	2.86	0.81
Simple sugars (g/kg/BM/day)	1.19	1.52
Crude fat (CF, g/kg BM/day)	0.60	0.44
Neutral detergent fibre (NDF, g/kg BM/day)	11.66	14.30
Acid detergent fibre (ADF, g/kg BM/day)	7.18	9.89
Calcium (mg/kg BM/day)	77	175
Phosphorus (mg/kg BM/day)	56	54

BM, body mass.

### 2.4 Measurements

Performance, gas exchange at exercise, and morphological changes were assessed regularly during the study (Figure 1).

#### 2.4.1 Body measurements

The horses were weighed after the morning meal every second week on a horse weighing scale (Tru-Test Eziweigh, Maréchalle-Pesage, France). The same day, the body condition was evaluated on a 0–5 scale (Arnaud et al., 1997), and the thicknesses of the *gluteus medius* (GM), at one-third of the distance from the tip of the hip to the tail head, were recorded by ultrasonography (Xario XG ultrasound scanner, Toshiba, Japan). The same manipulator trained to ultrasonography performed all the measurements with the horses positioned identically, all four limbs on the ground. The probe (PVT-375BT, Toshiba, Japan) was positioned orthogonally (90°). It was then tilted slightly (≤2°) to optimise echo-intensity without changing the muscle thickness measurement (Dankel et al., 2020). Ultrasound images were analysed using the software ImageJ by the same observer in a random order.

#### 2.4.2 Interval training and the 2,400 m performance test

During some IT sessions (W1, W3, W6, and W8), horses were equipped with heart rate monitors, which also collected GPS data (Equimetre, Arioneo, France). For the 2,400 m performance tests carried out on weeks 0, 3, 6, and 8, the rider wore a GPS watch (Forerunner 500, Garmin, France) to record the speed continuously and the time required to complete the distance.

#### 2.4.3 Incremental continuous exercise test

The ICET performed on W0 and W9 was preceded by a warm-up on the path at walk for 15 min, followed by a trot for 3 min at 4.2 m.s^−1^. Then, the horses started their test with 3 min of trotting at 5.6 m.s^−1^ on the pathway that leads to the track. Once on the track, the rider was instructed to increase the speed by 1.4 m.s^−1^ (5 km.h^−1^) every 2 minutes in successive stages until the horses could no longer maintain the requested speed at trot. The speed of the last completed stage was considered the maximum aerobic speed (MAS) at trot.

During the ICET, horses were equipped with a heart rate monitor that also collected GPS data in order to follow the actual speed at each stage (Polar Equine H10, Kemple, Finland). In addition, horses wore an Equimask (Cosmed, Italy) linked to a portable gas analysis system (K4b^2^, Cosmed, Italy) to monitor respiratory parameters. These included pulmonary ventilation ( $\dot{V}E$ ), amount of oxygen consumed ( $\dot{V}O_{2}$ ), and amount of carbon dioxide produced ( $\dot{V}{CO}_{2}$ ). Before each test, the O_2_ and CO_2_ analysers were calibrated as described by Art et al. (2006) using a two-point calibration with ambient air first and a gas of known concentration (O_2_: 16% and CO_2_: 5%). The delay between airflow and gas signals was also calibrated before each test with the sampling line used during the test. Finally, the rider wore a GPS watch (Forerunner 500, Garmin, France) to adjust and record instantaneous trotting speed.

### 2.5 Data analysis

#### 2.5.1 Data processing

During the IT sessions, maximal peak velocity (V_peak_) achieved at trotting was recorded. The linear acceleration from 5.0 m.s^−1^ to V_peak_ was calculated as the rate of change of the velocity in the given duration to reach V_peak_. The maximal acceleration per horse and training session was used for statistical analysis.

The average speed during the 2,400 m test was calculated from the time needed to cover the distance. In addition, the maximum heart rate value (HR_peak_) achieved during the test was also measured.

Data recorded during the ICET with the gas analysis system were downloaded using the K4b^2^ management data software (Cosmed, Italy). Breath-by-breath data were filtered with a 5-s average filter, and data were filtered for abnormalities on the K4b^2^ software. Data were then transferred to Excel files to convert $\dot{V}E$ , $\dot{V}O_{2}$ and $\dot{V}{CO}_{2}$ into mL.kg^−1^.min^−1^ to calculate the respiratory exchange ratio (RER) and the quotients of $\dot{V}E/\dot{V}{CO}_{2}$ and $\dot{V}E/\dot{V}O_{2}$ . The different stages of the test were identified from data points. Cardiorespiratory values were calculated as the averages over the last minute of each stage. Additionally, peak data achieved during the test were recorded during the final stage of the test, even if the stage was incomplete.

Whole body substrate oxidation was determined by calculating carbohydrate oxidation (CHOox) and lipid oxidation during exercise using the following equations (Frayn, 1983):

$$CHOox\;\left(g{.\min}^{-1}\right)=4.585\times {\dot{V}CO}_{2}-3.2255\times {\dot{V}O}_{2},$$

$$Lipid\;oxidation\;\left(g{.\min}^{-1}\right)=1.7012\times {\dot{V}O}_{2}-1.694{\times \dot{V}CO}_{2},$$

where $\dot{V}O_{2}$ and $\dot{V}{CO}_{2}$ are expressed in litres per minute. We assumed that protein oxidation made negligible contribution to $\dot{V}O_{2}$ and $\dot{V}{CO}_{2}$ in horses and that these equations developed for use in humans were also applicable to the horse (Geor et al., 2000). Then, CHOox and lipid oxidation in g.min^−1^ were converted to micromoles per kilogram body mass per minute. For CHOox, values expressed in g.min^−1^ were divided by the molecular weight of glucose (180.16 g.mol^−1^) and the horse’s body mass. For lipid oxidation, values in g.min^−1^ were divided by the molecular weight of palmitate (256.43 g.mol^−1^) and the horse’s body mass.

#### 2.5.2 Statistical analysis

Statistical analysis was performed with SAS software (SAS Studio, SAS Institute Inc., United States). The effects of diet, week, and the interaction diet * week on changes in BM, body condition score, thickness of the GM, maximal peak speed and acceleration measured during IT, average speed and HR_peak_ measured during the 2400 m tests, MAS, and cardiorespiratory variables (HR, $\dot{V}E$ , $\dot{V}O_{2}$ , $\dot{V}{CO}_{2}$ , RER, $\dot{V}E/\dot{V}{CO}_{2}$ , $\dot{V}E/\dot{V}O_{2}$ , CHOox, and lipid oxidation) measured during ICET were assessed using mixed-effect models with a normal distribution of errors in a MIXED procedure. The model included the horse as a random effect and weeks as repeated measures. For respiratory variables recorded at each stage during the ICET, the effect of speed and the interaction between speed and diet were also included in the MIXED model. Means were compared using the pdiff option in the LSMeans statement. Additionally, 95% confidence intervals (95% CI) were calculated for all means. Speed and cardiorespiratory variables recorded during the ICET were correlated using the CORR procedure. The level of significance was set at *p* ≤ 0.05.

## 3 Results

### 3.1 General considerations

All horses ate their entire rations without feed refusals, and no adverse effect on the health of the high-starch or alfalfa-rich diets was observed during the study. One horse from the group CON did not follow the training instructions from the second week of training, and its data were removed for analysis. For all the other horses, the training program, including the number of sessions and intensity, was fully respected all along the trial.

### 3.2 Body measurements

**FIGURE 2:**
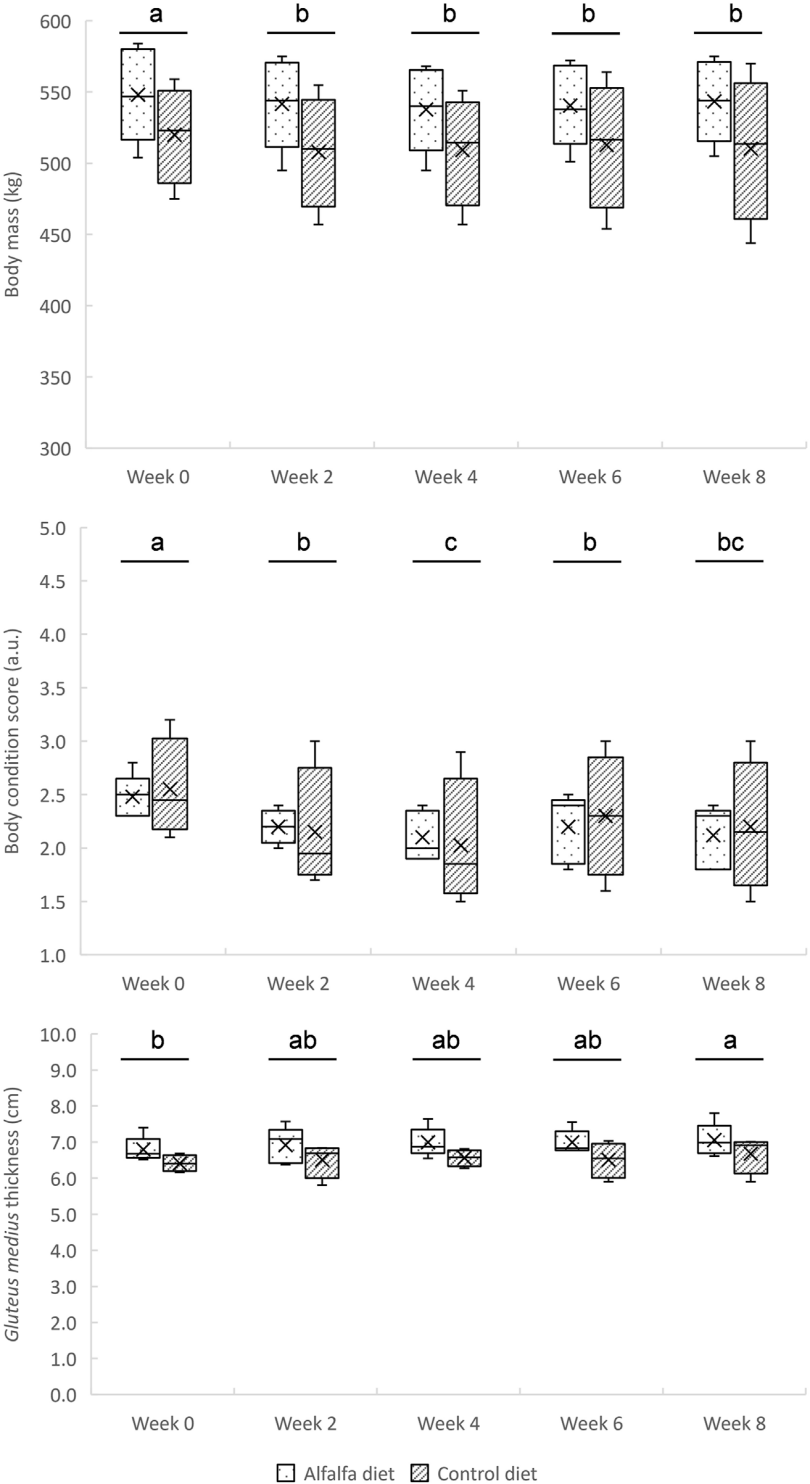
Changes in body mass, body condition score, and *gluteus medius* thickness during the 8-week training in horses fed a control diet or alfalfa diet. Mean values are reported. ^a, b, c^Superscripts indicate different (*p* < *0.*05) means between weeks.

Regardless of the diet (*p* = 0.250), the horses lost weight during the first 2 weeks of training and then maintained a stable weight until the end of the training period (*p* = 0.004) (Figure 2). Their body condition score also decreased until the fourth week of training before stabilising, (*p* < 0.0001) without effect of the diet (*p* = 0.930) (Figure 2). An increase in *gluteus medius* thickness was observed throughout the training period (*p* = 0.039), without difference between the groups CON and ALF (*p* = 0.150) (Figure 2).

### 3.3 Performance during IT and the 2,400 m test

**FIGURE 3:**
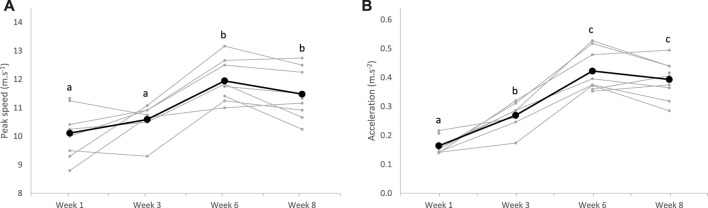
Maximal peak speed **(A)** and acceleration **(B)** measured during the interval training sessions over the 8-week training (bold lines represent means, and individual values are represented in light); ^a;b;c^superscripts indicate different (*p* < 0.05) means between weeks.

During recorded IT sessions, the maximal peak speed increased after 6 weeks of training (*p* < 0.0001), while acceleration improved since the third week of training (*p* < 0.0001), without any dietary effect (*p* = 0.257 and *p* = 0.191, respectively) (Figure 3).

**FIGURE 4:**
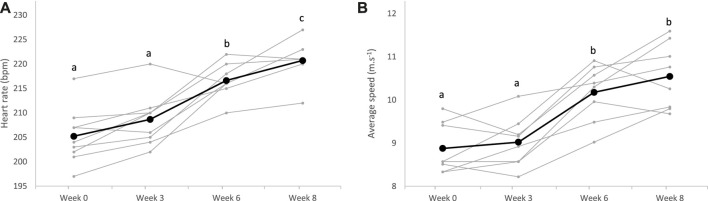
Heart rate peak **(A)** and average speed **(B)** measured during the 2,400 m test over the 8-week training (bold lines represent means, and individual values are represented in light); ^a;b;c^superscripts indicate different (*p* < 0.05) means between weeks.

Regarding the 2,400 m performance test, the HR_peak_ of horses significantly increased from the sixth week of training (*p* < 0.0001), with no effect of the diet (*p* = 0.565). Average speed on the 2,400 m concomitantly increased from the sixth week of training (*p* < 0.0001), and after the whole training period, horses covered the distance faster (8.9 m.s^−1^, 95% CI: 8.5–9.3 m.s^−1^) than at the beginning (10.6 m.s^−1^, 95% CI: 10.0–11.1 m.s^−1^), without difference between the groups CON and ALF (*p* = 0.130) (Figure 4).

### 3.4 Performance and cardiorespiratory responses during the ICET

MAS and HR_peak_ were significantly higher during the last stage in W9 compared to W0, without any significant effect of diet (Table 2). Regarding respiratory parameters, although peak $\dot{V}E$ , $\dot{V}O_{2}$ , and $\dot{V}{CO}_{2}$ were numerically higher after the training period, the differences were not significant (*p* = 0.158, *p* = 0.147, and *p* = 0.116, respectively).

**TABLE 2:** Effect of the diet and week on peak values during the ICET.

	Means value (LSMeans) and 95% CI for both groups		SEM	Effects (*p*-value)		
	Week 0	Week 9		Week	Diet	Week*Diet
MAS (m.s^−1^)	9.57^a^	10.53^b^	0.26	0.030	0.991	0.767
	9.40–9.74	9.88–11.17				
HR_peak_ (bpm)	215^a^	221^b^	2	0.007	0.257	0.088
	213–217	216–225				
$\dot{V}E_{peak}$ (mL.kg^−1^.min^−1^)	3,118	3,322	171	0.158	0.950	0.710
	2,908–3,434	2,981–3,660				
$\dot{V}O_{2peak}$ (mL.kg^−1^.min^−1^)	120	132	7	0.147	0.407	0.102
	109–136	119–148				
$\dot{V}{CO}_{2peak}$ (mL.kg^−1^.min^−1^)	121	132	6	0.116	0.394	0.863
	114–132	120–146				
RER_peak_	1.16	1.07	0.06	0.322	0.502	0.738
	1.01–1.32	1.01–1.14				

ICET, incremental continuous exercise test; MAS, maximum aerobic speed; HR, heart rate; $\dot{V}E$ , pulmonary ventilation; $\dot{V}O_{2}$ , amount of oxygen consumed; $\dot{V}{CO}_{2}$ , amount of carbon dioxide produced; RER, respiratory exchange ratio.

^a, b^LSMeans within a row differ significantly when superscripts are different (*p* < 0.05). Significant *p*-values are in bold.

Horses maintained a pace close to the setpoint for the speeds of each stage, as actual speeds averaged 5.1 ± 0.4, 7.0 ± 0.3, 8.4 ± 0.3, and 9.7 ± 0.3 m.s^−1^ for the four stages.

**FIGURE 5:**
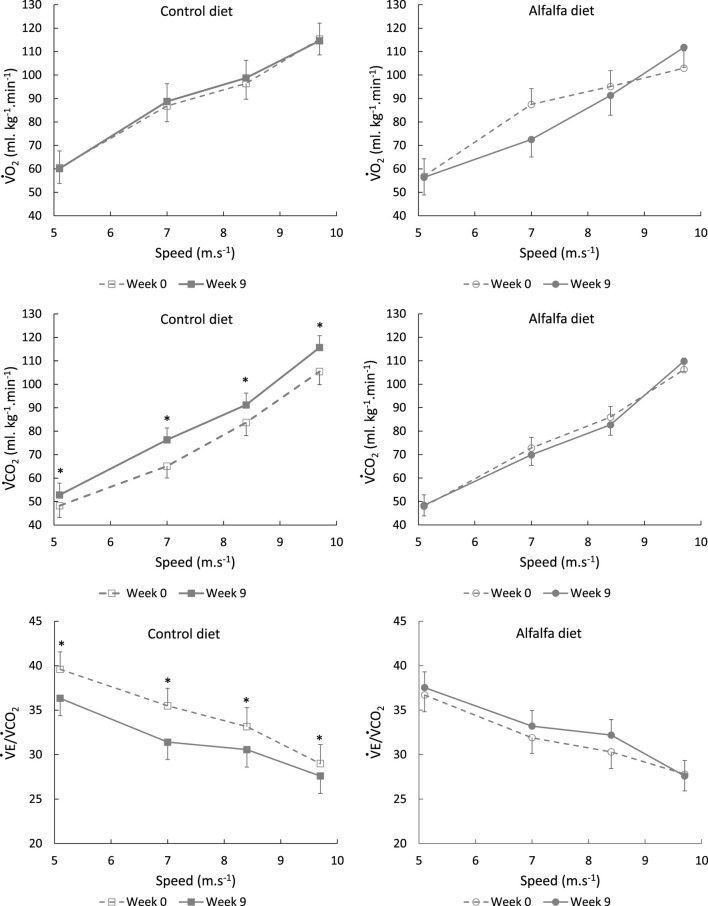
Average values (LSMeans and SEM) of $\dot{V}O_{2}$ , $\dot{V}{CO}_{2}$ , and ratio between $\dot{V}E$ and $\dot{V}{CO}_{2}$ during the incremental continuous exercise test depending on the speed stage in horses fed a control diet or alfalfa diet; *significant (*p* < 0.05) differences within the speed stage between weeks 0 and 9.

**FIGURE 6:**
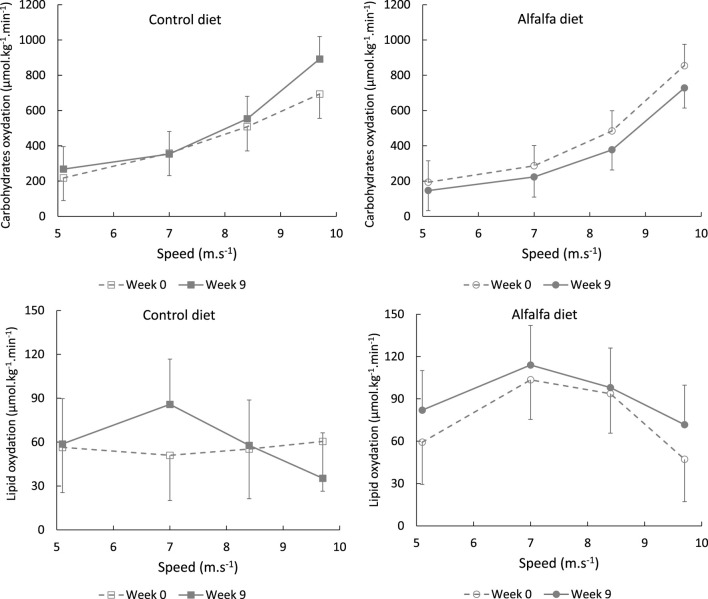
Average values (LSMeans and SEM) of carbohydrate and lipid oxidation during the incremental continuous exercise test depending on the speed stage in horses fed a control diet or alfalfa diet.

All averaged cardiorespiratory parameters were significantly influenced by the stage speed, with no significant difference between the groups CON and ALF. Heart rate, $\dot{V}E$ , $\dot{V}O_{2}$ , and $\dot{V}{CO}_{2}$ (Figure 5) increased between each stage from 5.6 to 9.7 m.s^−1^, while RER only increased from stage 8.3 m.s^−1^ (Table 3). The ratio $\dot{V}E/\dot{V}O_{2}$ differed between the first stage and the next ones, and $\dot{V}E/\dot{V}{CO}_{2}$ decreased gradually from the first to the last stage. A positive correlation was measured between speed and HR (r = 0.96, *p* < 0.0001), $\dot{V}E$ (r = 0.84, *p* < 0.0001), $\dot{V}O_{2}$ (r = 0.80, *p* < 0.0001), and $\dot{V}{CO}_{2}$ (r = 0.92, *p* < 0.0001). A significant increase in CHOox was also observed with speed (*p* < 0.0001) but lipid oxidation did not differ significantly between stages (*p* = 0.151) (Figure 6).

**TABLE 3:** Effect of diet, week, and speed on cardiorespiratory parameters and substrate oxidation per stage during the ICET.

	Means (LSMeans)		SEM	Means (LSMeans) and 95% confidence interval				SEM	Effects (*p*-value)				
	Week 0	Week 9		Stage 1 (5.6 m.s^−1^)	Stage 2 (7.0 m.s^−1^)	Stage 3 (8.3 m.s^−1^)	Stage 4 (9.7 m.s^−1^)		Week	Diet	Speed	Week* diet	Speed* diet
HR (bpm)	178^a^	182^b^	2	149^a^ 145–153	171^b^ 167–176	192^c^ 188–196	208^d^ 205–211	2	0.028	0.470	<.0001	0.568	0.712
$\dot{V}E$ (mL.kg^−1^.min^−1^)	2,450	2,505	108	1843^a^ 1728–1960	2329^b^ 2,243–2,502	2700^c^ 2,516–2,906	3037^d^ 2,838–3,290	113	0.248	0.653	<.0001	0.617	0.909
$\dot{V}O_{2}$ (mL.kg^−1^.min^−1^)	84	90	4	58^a^ 54–63	84^b^ 77–89	95^c^ 89–103	111^d^ 102–122	4	0.071	0.889	<.0001	0.594	0.699
$\dot{V}{CO}_{2}$ (mL.kg^−1^.min^−1^)	77	81	3	49^a^ 47–53	71^b^ 68–74	86^c^ 82–91	109^d^ 103–117	3	0.057	0.690	<.0001	0.031	0.902
RER	0.93	0.90	0.04	0.86^a^ 0.81–0.91	0.86^a^ 0.82–0.91	0.92^b^ 0.85–0.99	1.01^c^ 0.92–1.09	0.4	0.178	0.751	<.0001	0.131	0.654
$\dot{V}E/\dot{V}O_{2}$	46^b^	39^a^	2	47^b^ 42–51	40^a^ 37–43	42^a^ 37–45	40^a^ 36–44	2	<.0001	0.833	0.005	0.829	0.436
$\dot{V}E/\dot{V}{CO}_{2}$	33	32	1	38^c^ 35–40	33^b^ 31–35	32^b^ 30–33	28^a^ 26–29	2	0.185	0.718	<.0001	0.010	0.998
CHOox (µmol.kg^−1^.min^−1^)	450	443	71	207^a^ 154–276	306^a,b^ 232–399	481^b^ 369–613	793^b^629-964	77	0.865	0.631	<.0001	0.061	0.815
Lipid oxidation (µmol.kg^−1^.min^−1^)	66	76	17	64 45–81	89 61–121	76 51–113	54 23–87	18	0.393	0.432	0.151	0.593	0.674

ICET, incremental continuous exercise test; HR, heart rate; $\dot{V}E$ , pulmonary ventilation; $\dot{V}O_{2}$ , amount of oxygen consumed; $\dot{V}{CO}_{2}$ , amount of carbon dioxide produced; RER, respiratory exchange ratio; CHOox, carbohydrates oxidation.

^a, b, c, d^LSMeans within a row differ significantly when superscripts are different (*p* < 0.05). Significant *p*-values are in bold.

Compared to the basal values measured before training, the average HR during the test increased after the training period, and the $\dot{V}E/\dot{V}O_{2}$ ratio decreased. Although non-significant, average $\dot{V}O_{2}$ and $\dot{V}{CO}_{2}$ tended to be higher after the 8 weeks of training (*p* = 0.071 and *p* = 0.057, respectively).

Before training, $\dot{V}{CO}_{2}$ was similar between groups (*p* = 0.614) for the different speeds, but the changes in $\dot{V}{CO}_{2}$ after training differed between CON and ALF. Indeed, $\dot{V}{CO}_{2}$ did not change after training in the group ALF, while it increased after training for CON (*p* = 0.008) (Figure 5). Similarly, the ratio $\dot{V}E/\dot{V}{CO}_{2}$ that was similar between groups at W0, was not significantly different at W9 in group ALF (*p* = 0.323) but was lower at W9 in group CON (*p* = 0.009).

## 4 Discussion

### 4.1 General considerations

For the high-fibre group, it was decided to substitute 75% of the dietary cereals with dehydrated alfalfa, which contains more than 35% of digestible fibres. Alfalfa is a feedstuff commonly distributed to racehorses (Gallagher et al., 1992; Southwood et al., 1993; Sommaire, 2007; Kaya-Karasu et al., 2018). In addition to being a fibre-rich source of energy, trainers offer alfalfa to their exercised horses for its high acid buffering capacity (Jasaitis et al., 1987; Giger-Reverdin et al., 2002; Grimm et al., 2019), which can be beneficial to digestive health (Nadeau et al., 2000). Finally, some trainers select alfalfa for its high protein concentration, aiming to promote muscle synthesis (Matsui et al., 2006). In our study, the alfalfa-containing diet (ALF) provided 30% more proteins than the control diet (CON), while the energy supply was the same between the diets (Table 1).

During the first 2 weeks of training, the horses lost weight before stabilising without any effect of the diet (Figure 2). As horses were inactive before W0, it was expected that resumption of physical activity would induce a loss of weight and body condition (Garland et al., 2023). In some studies, it has been observed that horses fed forage-rich diets gained weight compared to concentrate-rich diets (Ellis et al., 2002; Jansson and Lindberg, 2012). It has been hypothesised that weight gain on high-forage diets could be due to increased DM and water intake and to longer mean retention time in the digestive tract (Ellis et al., 2002). This led to higher HR values during exercise (Ellis et al., 2002). In our study, the evolution of weight seemed instead associated with the resumption of training than with dietary management. The similar evolution between the groups may be due to the iso-energy intakes between diets, and to the slight differences in DM intake, as only part of the dietary cereals was replaced with dehydrated alfalfa (Table 1).

Although this was technically challenging and more time consuming, we conducted all riding training and exercise tests in field conditions on a track and not on a treadmill. Indeed, the physiological and mechanical results collected from the treadmill or racetrack tests can differ, even though running intensities are similar (Barrey et al., 1993; Couroucé et al., 1999). This has been related mainly with the difference in the rider or sulky load, ground surface effect, and movement between tracks and treadmill, which leads to alterations in muscle fibre recruitment and breathing patterns.

Field tests offer an accurate picture of horses’ cardiorespiratory patterns and energy metabolism in their everyday training environment, including loading a rider (van Erck et al., 2007). However, standardisation during field tests must be a major concern to minimise variability associated with the rider, the weather, and intensity instructions. In our trial, horses followed a training program that included three types of exercises. Two riders were involved in this training programme: one was in charge of all the interval trainings, one of the two other exercises, and all the performance tests. This was set up to avoid any rider effect on the measurements. Furthermore, all exercise tests were performed in early morning with dry weather conditions to minimise the influence of environmental conditions, which can interact with physiological responses (Hargreaves et al., 1999). Finally, the riders trained daily to monitor speed during exercises to maintain horses at the targeted intensities. This made it possible to respect the speeds imposed during the ICET strictly. Thanks to these standardised conditions, performance and cardiorespiratory data were comparable from one test to the other for each horse. The cardiorespiratory data we obtained were within expected ranges: HR ranged from 149 to 221 bpm depending on the intensity, $\dot{V}O_{2}$ between 58 and 132** **mL.kg^−1^.min^−1^, and $\dot{V}CO2$ between 49 and 132 mL.kg^−1^.min^−1^. We did not observe the underestimation of $\dot{V}{CO}_{2}$ reported during some field tests with trotters (van Erck et al., 2007), possibly because of the technical evolutions in CO_2_ concentration measurement in the K4b^2^ system.

To assess the performance and energy metabolism during exercise, horses were regularly monitored during some determined interval training sessions to measure their maximal speed and acceleration (Figure 1). In addition, two field tests were also set up and carried out before and after the 8 weeks of training. The first performance test was designed to mimic a 2,400 m race’s effort, a standard racing distance in Europe for trotters. The second test aimed at collecting cardiorespiratory data from the horses during an incremental running test performed until their maximum aerobic speed.

### 4.2 Effect of training

A significant increase in maximal peak speed and acceleration was observed during interval training from W1 to W6. Then, these values plateaued between W6 and W8 (Figure 3). In parallel to this riding test, the *gluteus medius* cross-sectional area was assessed by ultrasonography. This locomotor muscle was chosen because it is the heaviest pelvic limb muscle and is estimated to have the highest capacity for force and power among the proximal limb muscles (Payne et al., 2005). In addition, it is accessible to perform ultrasonography measurements and demonstrates an important response to exercise and training (Valberg, 2013). In our study, the *gluteus medius* thickness regularly increased in all horses throughout the 8 weeks of training (Figure 2). This result suggests that the implemented training, with repeated maximal accelerations, benefited muscle hypertrophy as demonstrated previously in Standardbreds who performed draft-loaded exercises (Gottlieb et al., 1989) or Thoroughbreds submitted to 12 weeks of conventional exercise training (Fitzharris et al., 2022). Although little used by equine trainers at present, this type of training with repeated maximum accelerations could be used to develop muscle mass in horses.

Over the training period, the average speed to run the 2,400 m at maximum intensity significantly improved in all horses (Figure 4). Between W0 and W3, progression was probably limited due to reaccustoming to work. The greatest improvement was observed between W3 and W6 when all horses significantly progressed. Afterward, progression was more variable between horses, and means plateaued. This rapid progression to a plateau after reaccustoming to work has already been described in previous reports in Standardbreds trained once daily (Shearman et al., 2002). To allow continued progression after W6, an increase in workload would probably have been necessary, possibly with two daily exercise sessions. Increasing the daily volume of training, with two sessions per day, is rarely practiced by horse trainers, generally due to lack of time. It would be interesting to study the impact of this increase, to determine the impact on equine performance. Along with speed, HR_peak_ also regularly increased with training during the 2,400 m tests (Figure 4). This was surprising as it is commonly accepted that maximum HR is not modified with training (Poole and Erickson, 2011). We suppose that as trotters evolved at a constrained gait, the peak HR we measured might not represent the maximum HR. With training, we assume that the maximal HR reached during the 2,400 m test approached the true maximum HR, mainly because the higher speed was reached at trotting (Hodgson, 2013).

After the 8-week training period, horses in both the groups increased their maximum aerobic speed during the ICET (Table 2), close to the results observed with French Trotters submitted to a similar harness exercise (Fortier et al., 2015). The peak heart rate increased to the same value as in the 2,400 m test, suggesting that the two tests demanded a maximal effort. This HR_peak_ seems consistent with previous data recorded in Standardbreds (Hodgson, 2013) and is close to the HR_peak_ measured during an ICET that was comparable in terms of track, speed, and stage duration (Fortier et al., 2015). However, no significant effect of training was observed on respiratory data at the end of the 8 weeks of training. Although non-significant, $\dot{V}E_{peak}$ , $\dot{V}O_{2peak}$ , and $\dot{V}{CO}_{2peak}$ numerically increased by 7%, 10%, and 9%, respectively, in both the groups (Table 2). A similar 10% increase in $\dot{V}O_{2peak}$ was described by Klein et al. (2020), with Standardbreds trained moderately for 12 weeks on a treadmill. It would be of interest to evaluate whether augmenting the frequency of high-intensity exercises could amplify the increase in $\dot{V}O_{2peak}$ in trotters. Regarding the cardiorespiratory responses at submaximal intensity, except for the HR, which was lower before the training period than after, the other parameters remained unchanged at every intensity stage (Table 3). The improved ventilatory equivalent for oxygen ( $\dot{V}E/\dot{V}O_{2}$ ratio) after training supports the improvement of performance. However, as $\dot{V}O_{2}$ was unchanged, this supposes that running economy was not altered with the training that was implemented.

### 4.3 Effect of diet

The 2,400 m running performance improved from W0 to W8 in both the groups irrespective of the diet (Figure 4), as previously observed in exercised trotters fed a high-energy forage providing 100% or 160% of the recommended protein intake (Connysson et al., 2006). The diet also had no effect on the increase in maximal peak speed and acceleration during IT (Figure 3). However, the detection of slight differences between the groups was limited by the sample size included in the trial. The comparable evolution of the athletic performance between the two groups might reflect the similar increase in muscle mass and maximal aerobic speed, regardless of the diet, although the diet ALF provided 30% more protein than the diet CON. Combining a high-protein diet with strength exercise has been associated with increased muscle synthesis in horses (Matsui et al., 2006). The latter authors observed that the muscle protein synthesis rate in the hindlimb was increased when an amino acid solution was perfused on horses immediately after a strength exercise. In our study, the concentrates were distributed at fixed times every day, but the duration between meal ingestion and exercise varied from 1 day to the next day. The augmentation in dietary protein might not have been sufficient to induce significant differences with the control group, or the delay between meal ingestion and exercise might not have been optimal to promote muscle synthesis with the high-protein diet. In addition, strength exercises were not daily in our experimental design, and a higher frequency could be required to increase muscle synthesis with protein intake.

Before the beginning of the experiment, all horses were fed a 100% hay diet. The average performance level and cardiorespiratory responses to exercise were similar in both the groups at W0 (Table 2; Table 3). During the training period from W1 to W8, one group received a control high-starch diet designed to mimic the diets usually distributed by trainers to exercised trotters, and one group received a diet where oats were mostly substituted for dehydrated alfalfa. The experimental diets were iso-energy, but the energy substrates differed between the control high-starch diet and the alfalfa high-fibre diet. The diet CON provided more starch and fewer fibres compared to the ALF diet, which was designed to theoretically promote fibre digestion and acetate production in the hindgut. After the 8-week training period, performance evaluated from the 2,400 m test had improved equally in both groups. However, the respiratory parameters measured during ICET differed between the horses fed high-starch or high-fibre diets. This suggests that the energy metabolism differed between the groups to achieve performance.

During the ICET performed at W9, oxygen consumption did not differ between the groups stage by stage (Figure 5). However, at W9, the horses fed the control diet exhaled more CO_2_ than those in the group ALF. Ventilation remained stable throughout the training period, resulting in a decreased $\dot{V}E/\dot{V}{CO}_{2}$ ratio at W9 for the group CON, while it remained stable for the group ALF. The rise in $\dot{V}{CO}_{2}$ probably reflected a modification of substrate utilisation during exercise. Although lipid oxidation was not altered, carbohydrate oxidation estimation tended to differ between groups at W9: CHOox increased from W0 to W9 for group CON, while it decreased for group ALF (Table 3). Our observations suggest that a high-fibre diet could limit glycolytic pathways in exercising horses. This confirmed previous results illustrating that the substitution of cereals by alfalfa lowers carbohydrate oxidation in horses exercised at 50% $\dot{V}O_{2\max}$ for 60 min (Jose-Cunilleras et al., 2002). However, the results related to carbohydrate and lipid oxidation should be used with caution in horses, as these results are calculated using equations which were developed in humans. Acetate, which is not considered in these equations, is much more important in horses and could bias the estimations. In this way, our data can be useful for future horse studies, to develop specific equations for this species.

Although we could not validate the modification in substrate utilisation with blood measurements or muscle biopsies during our trial, the metabolic modification we observed could be due to a greater availability of glucose with diet CON, either related to higher blood glucose or to higher glycogen storage. The modification could also be attributed to a higher availability of the other energy metabolites with diet ALF, especially acetate. As the diet CON provided more starch than the diet ALF, horses in the control group probably experienced higher glycaemia following concentrate meal ingestion than the horses fed alfalfa (Rodiek et Stull, 2007). In parallel, the high-fibre diet might have allowed greater digestion of fibres by the large intestine microbiota, which could have resulted in a higher blood concentration of acetate, as previously demonstrated (Jansson and Lindberg, 2012). In this latter work, blood acetate was about 50% higher in horses fed a high-fibre diet than that in horses fed a high-starch diet. Acetate is a direct precursor of the Krebs cycle under aerobic conditions and particularly well extracted from arterial blood in horses to contribute to oxidative metabolism (Pethick et al., 1993). The acetate uptake from the bloodstream, the transfer up to mitochondria, and the formation of acetyl-CoA from acetate do not produce protons (Moffett et al., 2020). On the contrary, glycolysis in muscles results in proton and pyruvate production. Protons produced during this process are buffered by sodium bicarbonate in the blood to maintain the acid–base balance and lead to higher CO_2_ production and exhalation during exercise (Péronnet and Aguilaniu, 2006; Lekeux et al., 2013). This could explain the difference in $\dot{V}{CO}_{2}$ that we observed in high-starch and high-fibre groups. This hypothesis is corroborated by previous lactate measurements: in Standardbreds, high-starch diets have been related to higher blood or intramuscular lactate during strenuous exercise compared to high-fibre diets (Karlsson et al., 2002; Jansson and Lindberg, 2012). This could reflect the higher mobilisation of glucose during physical activity in high-starch diets, required when acetate is less available. Thus, promoting the use of acetate in muscles, originating from fibre digestion, could reduce the use of the glycolytic pathway and delay the onset of metabolic acidosis. This opens perspectives in dietary management of athletic horses: not only is it essential to cover their energy needs but it also seems important to select diets that encourage the digestion of fibre by the intestinal microbiota.

## 5 Conclusion and perspectives

Our results illustrate that a high-fibre diet maintains running performance in French Trotter horses compared to traditional high-starch diets and suggest that it is possible to alter equine energy metabolism according to the selected diet. Promoting a high production of acetate by the intestinal microbiota could be a vector to modulate the metabolites used for muscle functioning. This could lead to a delayed onset of metabolic acidosis.

## Data Availability

The raw data supporting the conclusion of this article will be made available by the authors, without undue reservation.
